# Development and Implementation of Postdischarge Text Messages to Adolescents With Suicidal Thoughts and Behaviors Through Caring Contacts: Implementation Study

**DOI:** 10.2196/51570

**Published:** 2024-08-13

**Authors:** Glenn V Thomas, Elena Camacho, Fatimah A Masood, Yungui Huang, Jahnavi Valleru, Jeffrey A Bridge, John Ackerman

**Affiliations:** 1 Behavioral Health Services Nationwide Children’s Hospital Columbus, OH United States; 2 Department of Psychiatry & Behavioral Health The Ohio State University Columbus, OH United States; 3 The Abigail Wexner Research Institute Nationwide Children’s Hospital Columbus, OH United States; 4 Patient Safety Education & Research Ascension St. Louis, MO United States; 5 Department of Pediatrics The Ohio State University Columbus, OH United States

**Keywords:** mental health, suicide prevention, adolescent, caring contacts, mHealth, Zero Suicide, quality improvement, care transitions, safety plan, behavioral health, mobile phone

## Abstract

**Background:**

Youth suicide is a pressing public health concern, and transitions in care after a suicidal crisis represent a period of elevated risk. Disruptions in continuity of care and emotional support occur frequently. “Caring contacts” validating messages post discharge have the potential to enhance connections with patients and have been shown to improve outcomes. More recently, positive outcomes have been noted using caring contact text messages (SMS and MMS), which hold promise for engaging patients in a pediatric setting, but there are few studies describing the large-scale implementation of such an approach.

**Objective:**

This study aims to describe the process of developing and implementing automated caring contacts within a quality improvement framework, using a standardized series of supportive texts and images, for adolescents discharged from high-acuity programs at a large midwestern pediatric hospital. We describe lessons learned, including challenges and factors contributing to success.

**Methods:**

We implemented the caring contacts intervention in 3 phases. Phase 1 entailed developing supportive statements and images designed to promote hope, inclusivity, and connection in order to create 2 sets of 8 text messages and corresponding images. Phase 2 included piloting caring contacts manually in the hospital’s Psychiatric Crisis Department and Inpatient Psychiatry Unit and assessing the feasibility of implementation in other services, as well as developing workflows and addressing legal considerations. Phase 3 consisted of implementing an automated process to scale within 4 participating hospital services and integrating enrollment into the hospital’s electronic medical records. Process outcome measures included staff compliance with approaching and enrolling eligible patients and results from an optional posttext survey completed by participants.

**Results:**

Compliance data are presented for 4062 adolescent patients eligible for caring contacts. Overall, 88.65% (3601/4062) of eligible patients were approached, of whom 52.43% (1888/3601) were enrolled. In total, 94.92% (1792/1888) of enrolled participants completed the program. Comparisons of the patients eligible, approached, enrolled, and completed are presented. Primary reasons for eligible patients declining include not having access to a mobile phone (686/1705, 40.23%) and caregivers preferring to discuss the intervention at a later time (754/1705, 44.22%). The majority of patients responding to the optional posttext survey reported that the texts made them feel moderately to very hopeful (219/264, 83%), supported (232/264, 87.9%), that peers would be helped by these texts (243/264, 92%), and that they would like to keep receiving texts given the option (227/264, 86%).

**Conclusions:**

This study describes the successful implementation of automated postdischarge caring contacts texts to scale with an innovative use of images and demonstrates how a quality improvement methodology resulted in a more effective and efficient process. This paper also highlights the potential for technology to enhance care for at-risk youth and create more accessible, inclusive, and sustainable prevention strategies.

## Introduction

### Context

Youth suicide is a pressing public health concern and the third leading cause of death for individuals aged 10 to 19 years in the United States [1]. Suicide deaths among US youth increased during the COVID-19 emergency, with significantly more suicides than expected among male participants, non-Hispanic American Indian or Alaskan Native, and Black youth [2]. Regarding nonfatal suicidal behaviors, the percentage of youth presenting to pediatric hospitals for suicidal thoughts and attempts more than doubled between 2008 and 2015 [3]. A recent national study of emergency department (ED) visits in adolescents aged between 12 and 17 years reported significant increases in suicide attempts beginning in May 2020 and extending through March 2021, compared to corresponding periods in 2019 [4], with increases particularly elevated in females.

To address the needs of pediatric patients at risk of suicide more effectively, the behavioral health (BH) service line at our large Midwestern pediatric hospital adopted the Zero Suicide model, a comprehensive framework using a quality improvement (QI) approach to combine best practice tools and strategies to improve suicide care across health care systems with demonstrated reductions in patient suicides and suicidal behaviors [5-7]. Although identification and treatment of high-risk youth are core to Zero Suicide, transitions in care are also prioritized as vital points for system improvement. One potential means of improving outcomes for adolescents during care transitions is the use of caring contacts, now considered a best practice for transitions in care [8].

In this paper, we describe our hospital’s process of developing and implementing an automated and scalable universal caring contacts texting system for adolescents being discharged from the Psychiatric Crisis Department (PCD), Youth Crisis Stabilization Unit (YCSU), Inpatient Psychiatry Unit (IP), and Consult Liaison Service (CL) using an iterative QI approach to improve efficiency and enrollment of eligible patients and enhance health care equity with respect to age, sex, gender identity, race, and ethnicity.

### Problem Statement

While hospitalization or crisis care is often accessible for youth at imminent risk for suicide, the days and months after hospital discharge remain a high-risk period [9]. However, many youths receive limited (or no) follow-up mental health care [8,10-12], which is particularly concerning as follow-up care within 7 days of discharge is associated with a decreased risk for suicide [13]. Indeed, studies suggest that 42% of adolescents who attempt suicide do not attend their first scheduled appointment posthospitalization, and 25% do not attend even one follow-up session [10]. These concerning statistics underscore the need to maintain connections with high-risk patients as they transition levels of care, often across multiple, fragmented health care systems.

### Similar Interventions

Conceptualized by Motto [14] as validating, nondemand communications following a suicidal crisis, caring contacts are intended to enhance a patient’s inherent sense of self-worth and connectedness to ongoing supports [15]. Caring contacts originally took the form of personalized letters expressing concern without placing demands on the recipients (eg, no requests to engage in services or self-care). Working with a high-risk population of adult patients who refused, or did not follow through with, ongoing treatment upon discharge from a psychiatric facility, Motto and Bostrom [15] found significant reductions in suicide for those receiving caring contacts during the first 2 years post discharge. Of note, this reduction was no longer significant after 5 years, and the suicide rates for the intervention and control groups converged by year 14. The first year post discharge is the highest risk period for suicide and also saw the highest frequency of caring contact letters. Although effects may be time-limited, implementation of caring contacts has since been shown to reduce suicidal behaviors and readmissions across communication modalities (eg, letters, postcards, and phone calls), clinical populations, and cultures [16-25]. A recent meta-analysis [26] found that caring contacts reduced self-reported suicide attempts at 1 year but did not find significantly reduced suicide rates or ED visits and hospitalizations. However, the number of studies included in the analysis was small due to the limited number of available randomized controlled trials.

More recently, the feasibility and acceptability of texting as a means of communicating with caring contacts have been demonstrated in adults [19,27,28] and adolescents [29,30]. Furthermore, a randomized controlled trial demonstrated that a brief contact intervention delivered through text messages significantly reduced hospital-related self-harm visits over a 24-month period [31]. Using text messages has significant benefits: texts are nonintrusive, easily accessible, can be stored for later viewing, and the receiver has control over whether to remain involved. However, the existing literature exclusively features text-based caring contacts without the use of images. In considering the pediatric population served by our hospital, we decided to implement caring contact text messages paired with hopeful images to support adolescents during transitions of care as most youth have a high comfort level with receiving text messages [23]. The nearly ubiquitous use of smartphones by adolescents, even in households earning less than US $30,000 a year [32], and the high overall retention rate for youth involved in digital health interventions [33] presented an opportunity to text hopeful and engaging images along with supportive language in a confidential, nonstigmatizing format to all eligible patients without significant concern that access to a smartphone might be a barrier to equitable care.

## Methods

### Aims and Objectives

The main goal of this paper is to describe the development and implementation of a scalable automated text-based caring contacts intervention using a QI methodology to support eligible youth aged 13 to 18 years being discharged from acute services at our hospital following acute BH interventions related to a suicidal crisis. As a secondary objective, this implementation included a posttext survey to evaluate the extent to which patients found the texts hopeful and supportive.

### Blueprint Summary

#### Overview

In this study, caring contacts texts are supportive messages paired with hopeful images that include local and national youth crisis resources. Messages are unidirectional and do not serve as a text or chat service, although links are provided to youth who would like to access crisis support at any time.

There were three phases for this initiative: (1) phase 1: development and refinement of text messages (November 2018 to January 2019); (2) phase 2: piloting text messages and creating local workflows (February 2019 to February 2020); and (3) phase 3: full implementation with data collection across all 4 service areas (March 2020 to September 2021).

#### Phase 1: Development (November 2018 to January 2019)

The development of caring contacts message content began with the creation of validating, supportive statements and images aimed at promoting hope and a sense of inclusivity and connection to others while avoiding demanding or potentially emotionally intense or triggering content (Multimedia Appendix 1). An initial set of 16 images with supportive statements and information about crisis resources was developed by the hospital content experts in consultation with an adult former patient and member of the Zero Suicide implementation team, representing lived experience as a suicide attempt survivor. Messages were then reviewed by patient focus groups, representing diversity across race and gender, whose presenting concerns included suicidal ideation or behavior across inpatient and outpatient programs. Feedback from the focus groups indicated that the proposed texts were overwhelmingly viewed as positive and encouraging, were not emotionally triggering, and contained appropriate content. Feedback obtained from parent advocates, therapists, and suicide prevention experts also informed the development of 2 series of 8 caring contacts texts. Each text series was designed to be sent to individual patients over a 4-month period, starting the day after discharge, then weekly for 4 weeks, and monthly for the remaining 3 months. The second set of 8 texts was used for patients readmitted to the hospital.

#### Phase 2: Pilots (February 2019 to February 2020)

As part of our continuous QI efforts, each of the participating services collaboratively developed workflows to identify, consent or assent to receive texts, obtain contact information, and enroll eligible participants. Staff were then trained in these processes, and the first pilots were held, which consisted of multiple plan-do-study-act cycles [34] initially focusing on a small number of clinicians (between 1 and 5) enrolling between 1 and 4 patients each. Barriers to enrollment proved to be using a paper workflow rather than electronic medical records (EMRs), clinicians inconsistently approaching families for consent, failing to provide resources and enrollment forms, and forgetting eligibility criteria.

In response to these barriers, changes were made to the patient enrollment process, which was built directly into the EMR (completed in March 2020). We consulted with hospital legal services, and, given that caring contacts was now the standard care for youth with suicidal thoughts and behaviors, the hospital amended the general consent to allow for texting directly to adolescents aged 13 years and older, consistent with the Children’s Online Privacy Act.

**Figure 1:**
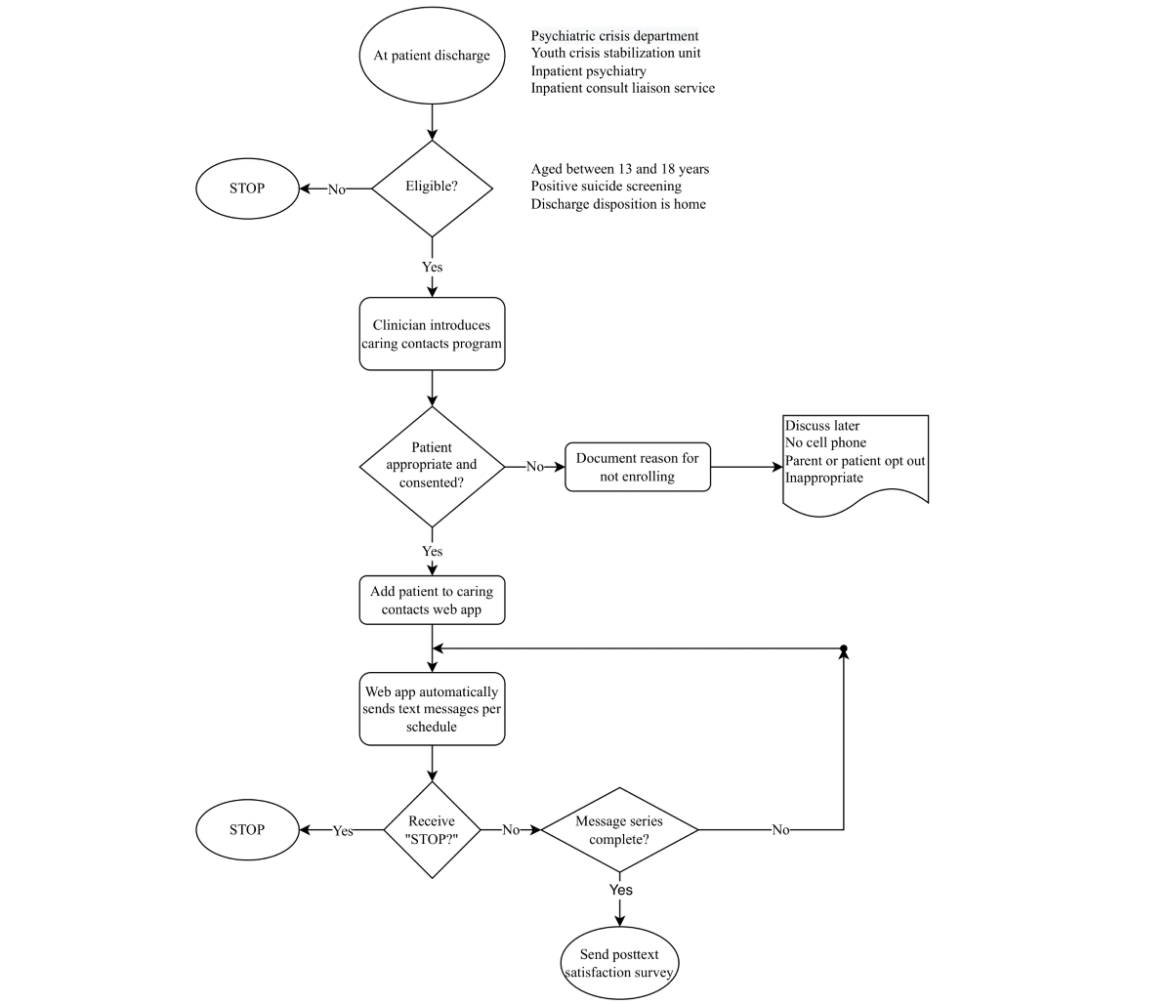
Process flow for the caring contacts program.

Early in phase 2, we hypothesized that the PCD would be the most effective site to enroll patients, as it serves as the point of entry for most hospital inpatient admissions to our system of care. However, low compliance rates and concerns that the discussion of care transition and caring contacts would fit better into the safety planning and discharge process led to the decision that each of the participating services would be responsible for enrollment at the time of a patient’s discharge. Toward the end of phase 2, all participating services were retrained in the new enrollment workflows. Figure 1 illustrates the final workflow.

#### Phase 3: Full-Scale Implementation With Data Collection (March 2020 to September 2021)

In March 2020, the responsibility for enrollment at the time of discharge was expanded to all participating services. A digital caring contacts enrollment form was implemented in the EMR, and we initiated automatic texting of the caring contacts through a web app. New participants were entered into the site as they were discharged from participating services, and their text sequence was initiated and scheduled automatically. In July 2020, the addition of a flag in the EMR reminding clinicians to enroll patients further increased the percentage of eligible patients approached.

### Process Measures

A QI approach for implementation places significant focus on compliance with established protocol to iteratively drive system changes to achieve its aims. One of the goals of this initiative was to maximize the enrollment of youth being discharged from the 4 participating services, making it imperative to identify and approach all eligible patients. Initially, eligible participants were identified through a visit diagnosis, billing diagnosis, or discharge diagnosis. However, in July 2019 during phase 2, BH implemented the Zero Suicide framework, including the use of a well-validated suicide screener at first contact for all patients with the Asking Suicide-Screening Questions [35] and, where appropriate, further risk assessment with the Columbia Suicide Severity Rating Scale [36]. Eligible participants were then identified based on a positive suicide risk endorsement on either measure.

A monthly compliance report was generated to assess adherence to the process, with two metrics, that were (1) percentage of eligible patients approached by staff to enroll and (2) percentage of eligible patients enrolled. Eligible participants were identified based on a positive suicide risk endorsement on the Ask Suicide-Screening Questions [35] and Columbia Suicide Severity Rating Scale [36] tools. For patients declining, 1 of 4 reasons was indicated by the clinician: no mobile phone, patient or family preferring to discuss enrollment at a later time, parent declined, and patient is developmentally inappropriate to enroll (comprehension challenges, autism spectrum, or nonverbal). The compliance data reported in this study were collected in phase 3, for which all participating services enrolled participants. In phase 3, due to the automation of the entire identification, approach, and enrollment process, accurate data could be obtained to determine compliance (compliance data are not reported for phase 2 due to the challenges in accurately determining the number of eligible patients for each plan-do-study-act, but actual numbers of the first texts sent in phase 2 are displayed in Multimedia Appendix 2). Summary compliance data were sent to the clinical leaders of the participating services to review and resolve program barriers to enrollment.

### Posttext Survey

Although focus groups suggested that automated texts and images were acceptable and affirming to patients, it was important to establish that this format was not problematic for participants and that they experienced a level of support and hopefulness by receiving them. A 4-question survey was sent to participants through a URL link in the final text. The survey used a Likert scale for the first two questions and yes or no for the last two questions: (1) On a scale of 1-5, how hopeful did these text messages make you feel? (2) On a scale of 1-5, how supported did these text messages make you feel? (3) Do you think other kids like you would be helped by these text messages? (4) If given the option, would you like to keep receiving text messages in the future?

### Data and Analysis

Data were extracted from 2 key sources—the EMR (ie, Epic) and the caring contacts web-based app database. Statistical process control methods were used to monitor compliance and analyze improvements over time. Specifically, c-charts allowed the study team to track the percentage of eligible patients approached and the percentage enrolled in caring contacts on a monthly basis for any special cause variation. Standard statistical process control rules were used to establish the control limits and identify special cause variation using the c-chart [37]. Tracking caring contacts enrollment compliance by unit was important as it allowed managers to provide feedback to individual clinicians and identify potential implementation barriers, which facilitated process improvement.

Psychiatric service areas and demographic factors (race, ethnicity, gender, and age) were retrospectively retrieved for all eligible patients based on the daily discharge list. Descriptive statistics of the demographic data and service areas for patients eligible for the program, patients approached about the program, patients enrolled in the program, and patients who completed the program, were generated. The approach rate is defined as the proportion of eligible patients who the clinical team approached to introduce the program. Enrollment rate is defined as the proportion of patients enrolled among patients approached, while completion rate is defined as the proportion of enrolled patients who completed the caring contact program fully. Point estimates of these rates were calculated for each demographic factor value and psychiatric service area, together with their corresponding 95% CIs. These data were examined in a pairwise fashion within each demographic factor or service area to see whether there were statistically significant differences in introduction, enrollment, and program completion rates. When the CIs of rates are nonoverlapping for a pair of factor values within the same category, such as 2 age groups or 2 different race values, then we can conclude the 2 factor values have statistically significant differences.

### Technical Design

In phase 2, all caring contacts texts were sent manually through a dedicated mobile phone. However, the large volume of texts per patient over time necessitated the development of an automated process through a web-based app. This required upfront investment but ensured scalability to additional hospital services, reduced the chances of human error, and reduced the administrative burden of a manual process, resulting in long-term savings.

The web-based app was developed to track patient enrollment, automatically send the programmed series of texts to participants, and update enrollees’ program progress and status. The app was built on an internal machine server behind the hospital firewall to maximize controls over security and privacy protection. Only patient phone numbers and nonpatient-specific message contents are passed to the messaging service provider, which stores the phone numbers for a limited time to ensure delivery. In turn, the hospital only receives the time of the message sent, the delivery status, and whether patients choose to opt out. Only hospital staff enroll patients to eliminate the inclusion of unnecessary details in the EMR. Youth attempting to reply to the text messages receive an auto-generated response validating their outreach and providing reminders of the different crisis options and links available around the clock.

### Target

This QI initiative addressed the use of caring contacts with high-risk adolescents between the ages of 13 and 18 years discharged from our highest acuity BH services at Nationwide Children’s Hospital, which serves as a regional hub for pediatric services in Ohio.

### Interoperability

The web-based app for caring contacts and the EMR system were designed to not communicate to protect patient health information and minimize risk.

### Participating Entities

A brief description of participating services is provided as follows: (1) PCD: a walk-in psychiatric ED for youth and families that serves as an entry point to higher levels of hospital-based care. (2) YCSU: crisis stabilization unit for voluntary admissions with a strong emphasis on individual and family therapy. (3) IP: inpatient psychiatry service with psychiatric unit milieu. (4) CL: psychiatric consult-liaison services provided to youth admitted for medical reasons who present with BH challenges during their admission.

### Budget Planning

Although grant-funded, startup costs for this initiative and resources were required to develop process flows and integrate these processes into the EMR. Some ongoing resources are also required to maintain the system. The overall budget for the development and maintenance of the caring contacts program was approximately US $102,560 across the 31-month study time frame. The research and development costs of US $27,720 included the technical design, creation of wireframes, application programming interface integration, testing, implementation, and maintenance to support the startup of automated caring contacts. Vendor fees were US $285 per month (US $3420 per year) throughout the study period. The cost of educational materials provided to families and staff was approximately US $300 per year. In addition, the management of caring contacts typically involves a percentage of the time of a bachelor’s-level staff member corresponding to program volume. Given the very high volume of acute care at this pediatric hospital, 50% of the effort was dedicated to personnel costs, equating to approximately US $25,250 per year and US $65,230 over the study period. Costs were covered by grant funding from the Ohio Suicide Prevention Foundation. In terms of replication, it is important to note that costs for new sites would be lower as startup and monthly fees are significantly lower due to a collaboration with a new vendor who offers startup to new sites using our design for US $2500 and a monthly text service fee of US $99 per month.

### Sustainability

Nationwide Children’s Hospital has made a significant commitment to reducing the pediatric suicide rate in the region and has significant resources devoted to data management and QI. Once grant funding for this initiative ends, we will allocate resources to support and sustain it.

### Ethical Considerations

The hospital’s Institutional Review Board (IRB) reviewed our proposal to implement caring contacts as a QI initiative and determined that this initiative did not constitute human subjects research under 45 Code of Federal Regulations part 46 or 21 Code of Federal Regulations part 50. Participants were not offered or provided with any form of compensation.

## Results

### Coverage

This initiative was implemented at Nationwide Children’s Hospital, the second-largest children’s hospital in the United States, which provides 38 licensed inpatient psychiatric beds and an additional 16 beds in the YCSU. Annually, it facilitates over 2000 discharges from these units. The PCD manages approximately 8000 visits each year. Furthermore, over 240,000 BH outpatient visits occur annually, underscoring its substantial regional role in pediatric mental health care.

### Outcomes

From March 2019 to September 2021, a total of 4062 adolescent patients were eligible to receive caring contacts text messages. The total number of youths approached for the caring contact program (n=3601), total number enrolled (n=1888), and total number of youths having completed the program (n=1792) are listed in Table 1, together with summary statistics breakdowns by demographic factors and psychiatric service areas for these patient cohorts.

**Figure 2:**
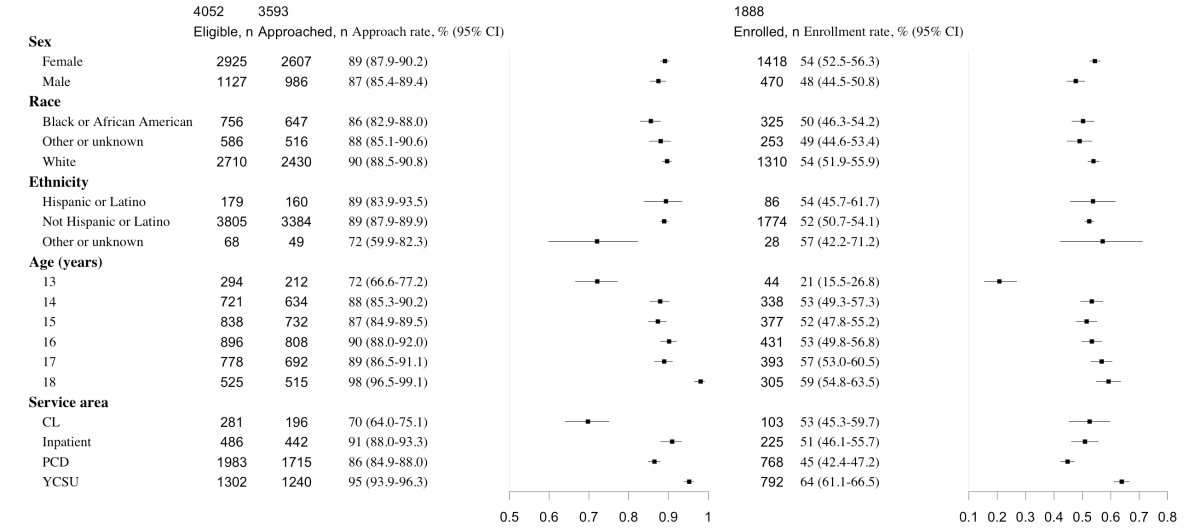
Comparison of patients eligible, approached, and enrolled. CL: Consult Liaison Service; PCD: Psychiatric Crisis Department; YCSU: Youth Crisis Stabilization Unit.

While compliance data for phase 2 could not be calculated, Multimedia Appendix 2 displays the actual number of texts sent by month during the pilots before automation. Once phase 3 began in March 2020, it took 3 months to consistently achieve an approach rate of over 90% (Multimedia Appendix 3). The enrollment rate by month is reflected in Multimedia Appendix 4. The overall patient approach rate for the 19-month period was 89% (95% CI 88-90), with an enrollment rate of 53% (95% CI 51-54), and a completion rate of 95% (95% CI 94-96; Figure 2). The table on the left of each panel in Figure 2 shows the total number of patients eligible, approached, enrolled, and completed, stratified by demographic factors and psychiatry service areas. The point estimates of approach rates, enrollment rates, and completion rates are listed, along with the associated CIs. Point estimates and 95% CIs of these rates are also visually represented on the right of each panel in Figure 2.

We assessed differences in age, gender, and race or ethnicity in an initial effort to identify whether approach rates were conducted equitably. There were no significant differences in approach rates between male and female patients. However, the approach rate for the White patient group (513/1189, 89.67%) was significantly higher than for the Black patient group (647/756, 85.6%). The approach rate for the patient group with another or unknown ethnicity was significantly lower than the other patient groups. The approach rate for the 13-year-old patient group was significantly lower than all other age groups, while the approach rate for the 18-years-old patient group was significantly higher than the other age groups. The pairwise approach rates were all statistically different between any 2 service areas in CL, PCD, IP, and YCSU, ordering from the lowest to the highest. The program enrollment data show that females enrolled in the caring contact program at a significantly higher rate than males (54% vs 48%). There were no significant differences in enrollment by race or ethnicity. The 13-year-old patient group had a significantly lower enrollment rate than older groups. YCSU had a significantly higher enrollment rate than the other service areas.

The reasons for declining the caring contact program are summarized in Table 2. The “no cell phone” option was chosen at a significantly higher rate for females (513/1189, 43.15%) than for males (173/516, 33.5%), while “inappropriate” was chosen at a significantly higher rate for males (55/516, 10.7%) than for females (65/1189, 5.5%). “No cell phone” was selected at a significantly higher rate for 13-year-olds (91/168, 54.2%) than for 16- (142/377, 37.7%), 17- (101/299, 33.8%), and 18-year-olds (71/210, 33.8%). “Inappropriate” was selected at the significantly highest rate for inpatient consultation or liaison, while “inappropriate” was selected at the significantly lowest rate for YCSU among all service areas. “Discuss at a later time” was selected at a significantly higher rate in the PCD (442/947, 46.7%) than the CL (30/93, 32%) and inpatient (79/217, 36.4%).

Program completion data indicated that females also completed the caring contact program at a significantly higher rate than males (1360/1418, 95.9% vs 432/470, 91.9%). There were no significant differences in program completion by race, ethnicity, age group, or service area.

**Table 1:** Patient cohort descriptive statistics.

Values		Eligible, n (%)	Approached, n (%)	Enrolled, n (%)	Completed, n (%)
**Sex**					
	Female	2925 (72)	2607 (73)	1418 (75)	1360 (76)
	Male	1127 (28)	987 (27)	470 (25)	432 (24)
**Race**					
	Black or African American	756 (19)	647 (18)	325 (17)	310 (17)
	Other or unknown	586 (14)	516 (14)	253 (13)	238 (13)
	White	2710 (67)	2430 (68)	1310 (69)	1244 (69)
**Ethnicity**					
	Hispano or Latino	179 (4)	160 (4)	85 (5)	79 (4)
	Not Hispanic or Latino	3805 (94)	3384 (94)	1774 (94)	1687 (94)
	Other or unknown	68 (2)	49 (1)	28 (1)	26 (1)
**Age (years)**					
	13	294 (7)	212 (6)	44 (2)	44 (2)
	14	721 (18)	634 (18)	338 (18)	326 (18)
	15	838 (21)	732 (20)	377 (20)	359 (20)
	16	896 (22)	808 (22)	431 (23)	405 (23)
	17	778 (19)	692 (19)	393 (21)	371 (21)
	18	525 (13)	515 (14)	305 (16)	287 (16)
**Service area**					
	CL^a^	281 (7)	196 (5)	103 (5)	96 (5)
	Inpatient	486 (12)	442 (12)	225 (12)	212 (12)
	PCD^b^	1983 (49)	1715 (48)	768 (41)	722 (40)
	YCSU^c^	1302 (32)	1240 (35)	792 (42)	762 (43)

^a^CL: Consult Liaison Service.

^b^PCD: Psychiatric Crisis Department.

^c^YCSU: Youth Crisis Stabilization Unit.

**Table 2:** Reasons for declining caring contacts.

Category			No cell phone		Inappropriate		Parent opt out		Discuss at a later time	
		Total, N	n (%)	95% CI	n (%)	95% CI	n (%)	95% CI	n (%)	95% CI
**Sex**										
	Female	1189	513 (43)^a^	40-46^a^	65 (5)^a^	4-7^a^	98 (8)	7-10	513 (43)	40-46
	Male	516	173 (34)^a^	29-38^a^	55 (11)^a^	8-14^a^	47 (9)	7-12	241 (47)	42-51
**Race**										
	Black or African American	322	140 (43)	38-49	22 (7)	4-10	25 (8)	5-11	135 (42)	36-47
	Other or unknown	263	96 (37)	31-43	20 (8)	5-12	27 (10)	7-15	120 (46)	39-52
	White	1120	450 (40)	37-43	78 (7)	6-9	93 (8)	7-10	499 (44)	41-47
**Ethnicity**										
	Hispanic or Latino	74	25 (34)	23-46	—^b^	—	—	—	—	—
	Not Hispanic or Latino	1610	650 (40)	38-43	—	—	—	—	—	—
	Other or unknown	21	11 (52)	30-74	—	—	—	—	—	—
**Age (years)**										
	13	168	91 (54)^a^	46-62^a^	—	—	—	—	58 (35)	27-42
	14	296	128 (43)	38-49	20 (7)	4-10	18 (6)	4-9	130 (44)	38-50
	15	355	153 (43)	38-48	26 (7)	5-11	23 (6)	4-10	153 (43)	38-48
	16	377	142 (38)^a^	33-43^a^	23 (6)	4-9	36 (9)	7-13	176 (46)	41-51
	17	299	101 (34)^a^	28-39^a^	22 (7)	5-11	35 (12)	8-16	141 (47)	41-53
	18	210	71 (34)^a^	27-41^a^	—	—	—	—	96 (45)	39-52
**Service area**										
	CL^c^	93	32 (34)	24-44	—	—	—	—	30 (32)^a^	22-42^a^
	Inpatient	217	99 (46)	39-53	21 (10)	6-14	18 (8)	5-13	79 (36)^a^	30-43.2^a^
	PCD^d^	947	366 (39)	35-42	69 (7)	6-9	72 (8)	6-9	442 (47)^a^	43.3-50^a^
	YCSU^e^	448	195 (43)	39-48	—	—	—	—	203 (45)	40-50
	Total	1705	686 (40)	38-43	120 (7)	6-8	145 (8)	7-10	754 (44)	42-46

^a^The proportion is statistically significantly different than at least another value in the demographic category, for example, proportions of “no cell phone” among females and males are statistically significantly different.

^b^Cell value of “—” is used to avoid the disclosure of cell counts less than 11.

^c^CL: Consult Liaison Service.

^d^PCD: Psychiatric Crisis Department.

^e^YCSU: Youth Crisis Stabilization Unit.

A total of 1961 optional posttext satisfaction surveys were sent, of which 264 (13.46%) were completed. A total of 219 (83%) respondents reported that the texts made them feel moderately to very hopeful, 232 (87.9%) respondents reported feeling moderately to very supported, 243 (92%) respondents reported that peers would be helped by these text messages, and 227 (86%) respondents reported they would like to keep receiving texts in the future if given the option.

### Lessons Learned

#### Success Factors

As expected for any large-scale implementation initiative, engaging staff across disciplines and hierarchy was a critical factor in successful implementation. Service areas with actively engaged leaders and champions facilitated smooth integration of the initiative into existing workflows and demonstrated greater adaptability and flexibility throughout the process. Embedding these new procedures seamlessly into the EMR and daily workflows of clinicians also contributed significantly to successful implementation.

#### Challenges

High volume periods, especially when combined with low staffing levels, negatively impacted compliance and may have contributed to clinicians checking the “discuss later” reason for declining the intervention instead of taking the time to educate and enroll families. Staff turnover and program workflow changes led to a need for regular updated training. Furthermore, the data suggest implicit bias may have impacted approach rates.

## Discussion

### Overview

This paper describes the process of developing and implementing automated caring contacts, containing both text and images, for adolescents receiving acute services in a large BH service line using a QI methodology, with the ultimate goal of decreasing suicide risk during transitions in care. We were able to demonstrate that an automated system can be delivered at scale and that data obtained from the system can be leveraged to provide rapid improvement in adherence to established protocols. Moreover, the addition of images to the texts appears to be acceptable to an adolescent population. Consistent with the initial implementation of caring contacts letters by Dr Jerome Motto, these texts and hopeful images were expressions of care and validation. However, using a pre-established series of texts for all patients deviates from the original concept of sending personalized letters. The low dropout rate and posttext survey results suggest this was not a barrier to our patients. Youths receiving caring contacts reported that they felt supported and hopeful and that they thought this intervention would help others. Not personalizing the content of texts ensured that communication minimized the potential for exposure to protected health information and allowed us to significantly expand capacity. This has improved efficiency and, arguably, the cost-benefit ratio of this initiative. Further analyses will examine whether there has been a reduction in return visits to the PCD and psychiatric readmissions and whether this varies by participating service unit.

Our intent was to scale up this initiative through an iterative QI process. This approach enabled us to refine enrollment procedures, seamlessly integrate the initiative into the EMR, and enhance recruitment strategies. As a result, we relatively quickly achieved over 90% of eligible patients being approached monthly, with a consistent enrollment rate exceeding 50%. The potential benefits of implementing caring contacts to scale are significant—decreased loss to suicide, decreased suicide contagion, improved hospital readmission rates, decreased costs, and decreased pressure on an already overburdened mental health system.

Our process outcomes reflected some significant differences by age group in approach, enrollment, and completion rates. The 13-year-old patient group was less likely to be approached and had a lower enrollment rate than the other age groups, which may be due to a lower rate of mobile phone access. The lower approach rate is also potentially a reflection of lower perceived autonomy and clinician perceptions of lower risk in younger patients. In general, the enrollment rate increased with age, and the 18-year-old group had a significantly higher enrollment rate than the younger age groups. In addition, females were more likely than males to enroll in and complete this initiative, possibly reflecting an increased openness to accessing mental health support and to emotional information processing.

Approach rates differed by service type. Specifically, the approach rate was higher for the YCSU than for other service areas, and their patients were also more likely to enroll than patients in the other services. The YCSU was also less likely to endorse the “discuss at a later time” reason for declining the intervention. These differences may reflect the unit’s relatively high acuity, focus on intensive therapy, including family therapy, favorable clinician-to-patient staffing ratios, and patient population (older youth admitted voluntarily and without significant disruptive behaviors). The CL service had a significantly lower approach rate than the other participating services and also had the highest use of the “inappropriate” decline reason. This likely reflects the nature of the CL service, which is spread across medical units rather than being on a single unit, and possibly lower engagement with the CL clinicians. As might be expected with a real-world QI implementation, there were external pressures that impacted staff compliance over time, such as high-volume periods that decreased the likelihood eligible patients would be approached.

We posited that a large-scale suicide prevention intervention made available to a broad population would increase health care equity as all eligible participants would be identified and approached. However, Black youth were less likely to be approached to participate than their White peers (647/756, 85.6% vs 2430/2710, 89.67%), possibly reflecting implicit bias on the part of clinicians. To further examine this difference by race, we analyzed the approach rates by race for each service area separately but found no statistically significant differences, which could be due to smaller cell sizes. There were no differences in enrollment or completion by race or ethnicity. The percentage of patients without access to a smartphone was higher than expected, but there were no statistically significant differences by race.

A limitation of this study is that demographic data were not collected for the focus groups, and it is unclear whether feedback was obtained at similar rates from youth of color as from White youth. However, the data reflect no significant differences in completion rates based on race or ethnicity. There were also no follow-up data collected for families who declined because they preferred to discuss the initiative at a later time. Another limitation was the relatively low rate of response to the survey and the potential for selection bias; our results may not be representative of all youth enrolled. As this implementation was a QI initiative rather than a research study, all data were aggregated, and there was no control group.

Based on the literature suggesting the year after discharge is a high-risk period, we moved to sending a set of 19 texts over the course of a year, starting in September 2021. Future enhancements include implementing caring contacts in other languages (Spanish, Somali, Arabic, and Nepali) to ensure cultural inclusivity. In addition, as the hospital expands the larger Zero Suicide framework across departments beyond the BH service line, we have also expanded caring contacts, starting with primary care and several intensive outpatient programs. We have begun the dissemination of our caring contacts content and processes to other health care systems in Ohio as well. Through this effort, we have standardized our processes and workflows, which will ultimately make implementation easier and more cost-efficient for our health care partners. Notably, during the initial manual phase, caregivers frequently expressed interest in receiving caring contacts and additional resources. This highlights the potential value of additional support for those caring for at-risk youth. Therefore, we are developing a set of caring contacts specifically tailored to support caregivers.

Future research could focus on identifying an optimal number and duration of texts and whether the addition of images impacts outcomes. As we plan to expand the program to include multilingual support and adapt to broader health care contexts, subsequent research will be essential in evaluating these adaptations’ effectiveness, the stability of outcomes across diverse groups, and ensuring that the interventions remain culturally sensitive and equitable. There is preliminary support suggesting that cultural adaptations to caring contacts are acceptable across diverse populations [38]. Furthermore, this study lays the groundwork for future studies to explore the long-term impacts of automated messaging on suicide prevention outcomes for youth, the nuances of its acceptability across different demographic groups, and its integration into various health care settings.

### Conclusions

In summary, this study describes the implementation of a scalable, automated caring contacts intervention integrated into a larger suicide prevention QI framework, in our case the Zero Suicide model, within a large health care system that is sustainable over time. An innovative component of our implementation was the use of images to engage participants. Consistent with other studies, our data suggest that this is an acceptable approach to intervening with adolescents at risk for suicide. The scalability and acceptability emphasize the potential for implementing automated supportive interventions on a broader scale, offering a viable pathway to enhancing postdischarge care for at-risk youth. This study highlights the growing potential for leveraging technology in mental health interventions and shifting toward more accessible, inclusive, and sustainable suicide prevention strategies.

## Appendix

Multimedia Appendix 1 Example of a Caring Contact text.

Multimedia Appendix 2 Number of first texts sent in phase 2 before automation.

Multimedia Appendix 3 Patients approached for caring contacts enrollment in phase 3.

Multimedia Appendix 4 Patients enrolled in caring contacts in phase 3.

## Abbreviations

BH: Behavioral Health
CL: Consult Liaison Service
ED: emergency department
EMR: electronic medical records
IP: Inpatient Psychiatry Unit
PCD: Psychiatric Crisis Department
QI: quality improvement
YCSU: Youth Crisis Stabilization Unit

## Data Availability

The data sets generated and analyzed during this study are available from the corresponding author on reasonable request.
