# Supplementary material for: Development and Implementation of Postdischarge Text Messages to Adolescents With Suicidal Thoughts and Behaviors Through Caring Contacts: Implementation Study
Source: JMIR Pediatr Parent. 2024 Aug 13;7:e51570. doi: 10.2196/51570 (PMC11350296; doi:10.2196/51570)
Supplement: Multimedia Appendix 1 [file pediatrics_v7i1e51570_app1.docx]

| 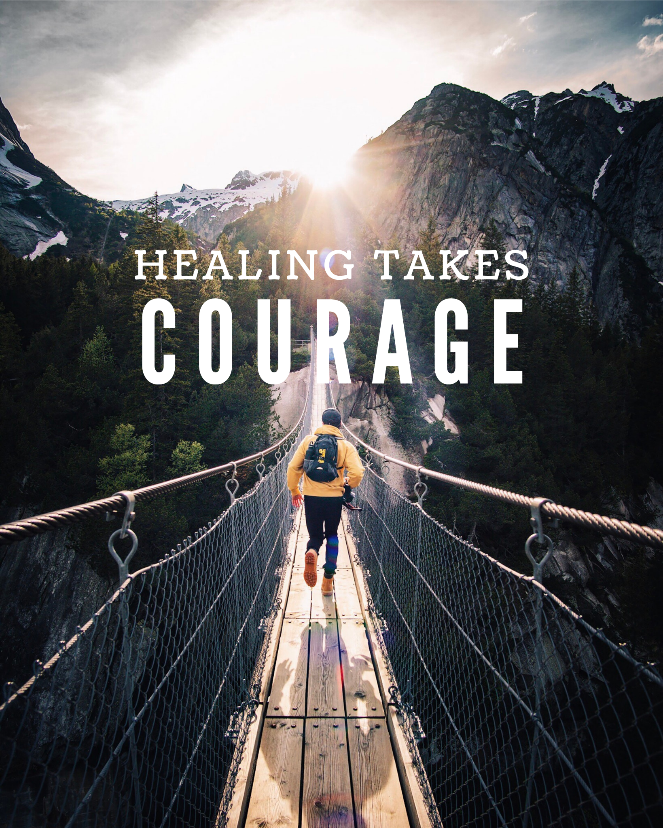  Healing takes courage - you've already taken the first steps by accepting help. You are stronger than you think.  These are not monitored, and we're unable to reply. If you're struggling, remember your safety plan or call the Youth Crisis Line at 614-722-1800 or text 4HOPE to 741741. For emergencies dial 911. Data and message rates may apply. You can reply STOP to opt out. |
| --- |
