# Supplementary figures and images for: Development and Implementation of Postdischarge Text Messages to Adolescents With Suicidal Thoughts and Behaviors Through Caring Contacts: Implementation Study
Source: JMIR Pediatr Parent. 2024 Aug 13;7:e51570. doi: 10.2196/51570 (PMC11350296; doi:10.2196/51570)

**Figure S1**


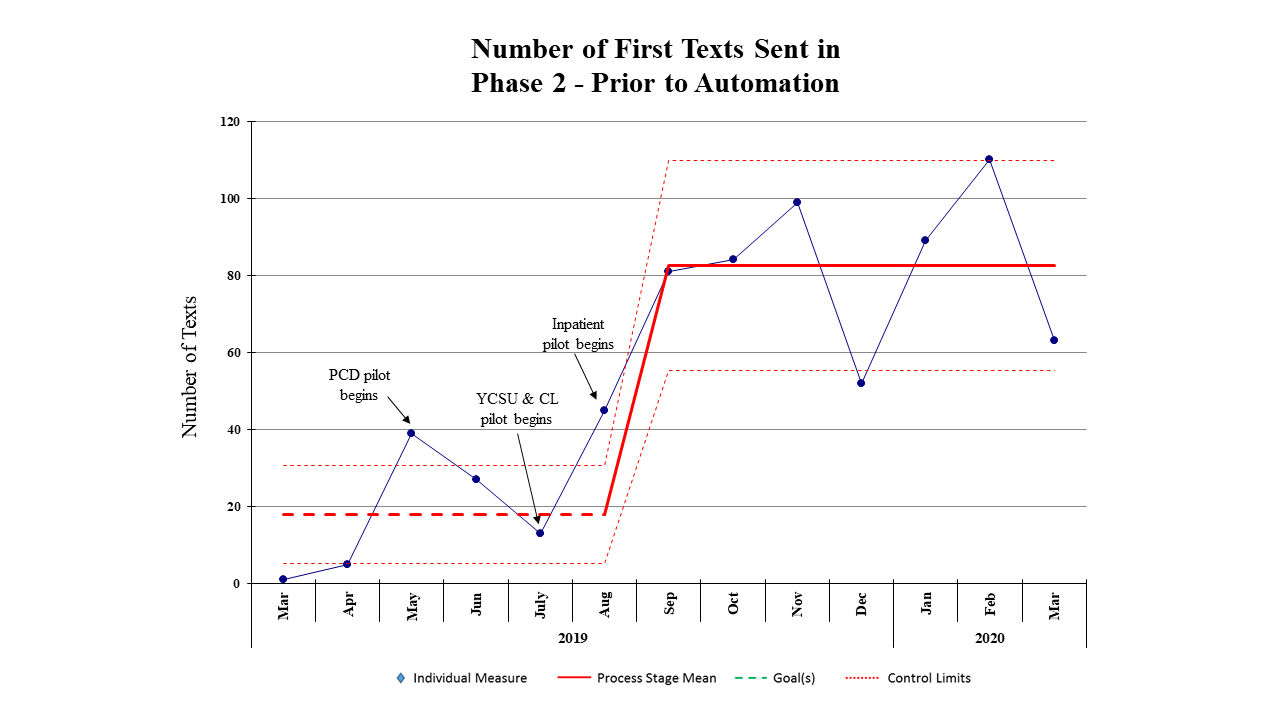

Supplement: Multimedia Appendix 2 [file pediatrics_v7i1e51570_app2.docx]

**Figure S1**


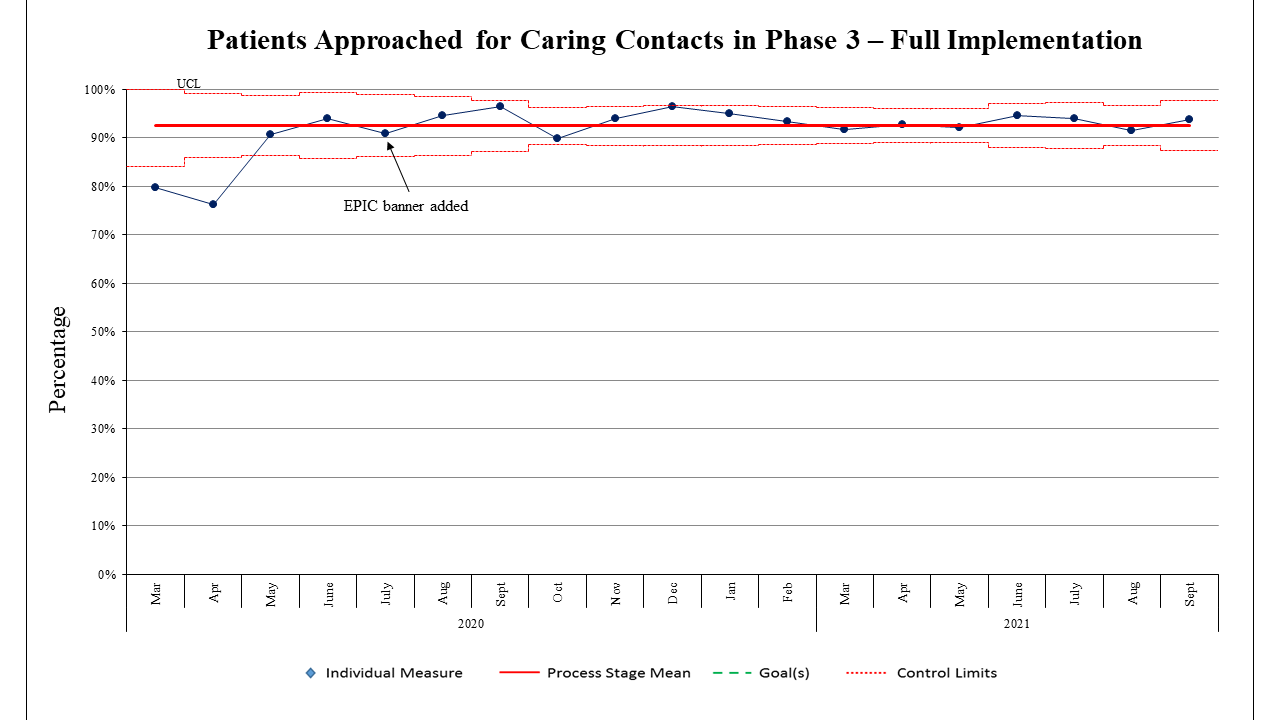

Supplement: Multimedia Appendix 3 [file pediatrics_v7i1e51570_app3.docx]

**Figure S1**


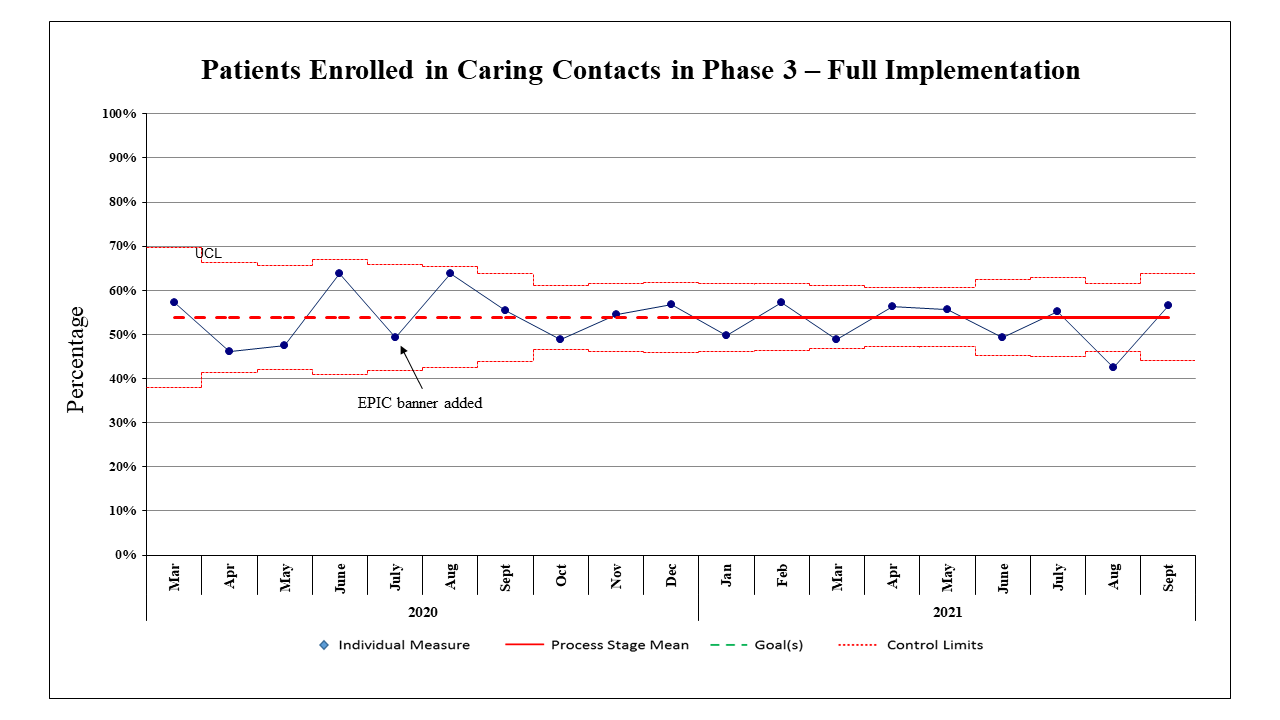

Supplement: Multimedia Appendix 4 [file pediatrics_v7i1e51570_app4.docx]
